# Whole Genome Sequencing of *Escherichia coli* From Store-Bought Produce

**DOI:** 10.3389/fmicb.2019.03050

**Published:** 2020-01-29

**Authors:** Cameron J. Reid, Khald Blau, Sven Jechalke, Kornelia Smalla, Steven P. Djordjevic

**Affiliations:** ^1^The ithree Institute, University of Technology Sydney, Ultimo, NSW, Australia; ^2^Julius Kühn-Institut, Federal Research Centre for Cultivated Plants, Institute for Epidemiology and Pathogen Diagnostics, Braunschweig, Germany; ^3^Institute for Phytopathology, Justus Liebig University Giessen, Giessen, Germany

**Keywords:** *E. coli*, produce, antimicrobial resistance, whole genome sequencing, genomic surveillance

## Abstract

The role of agriculture in the transfer of drug resistant pathogens to humans is widely debated and poorly understood. *Escherichia coli* is a valuable indicator organism for contamination and carriage of antimicrobial resistance (AMR) in foods. Whilst whole genome sequences for *E. coli* from animals and associated meats are common, sequences from produce are scarce. Produce may acquire drug resistant *E. coli* from animal manure fertilizers, contaminated irrigation water and wildlife, particularly birds. Whole genome sequencing was used to characterize 120 tetracycline (TET) resistant *E. coli* from store-bought, ready-to-eat cilantro, arugula and mixed salad from two German cities. *E. coli* were recovered on the day of purchase and after 7 days of refrigeration. Cilantro was far more frequently contaminated with TET-resistant *E. coli* providing 102 (85%) sequenced strains. Phylogroup B1 dominated the collection (*n* = 84, 70%) with multi-locus sequence types B1-ST6186 (*n* = 37, 31%), C-ST165 (*n* = 17, 14%), B1-ST58 (*n* = 14, 12%), B1-ST641 (*n* = 8, 7%), and C-ST88 (*n* = 5, 4%) frequently identified. Notably, seven strains of diverse sequence type (ST) carried genetic indicators of ColV virulence plasmid carriage. A number of previously identified and novel integrons associated with insertion elements including IS*26* were also identified. Storage may affect the lineages of *E. coli* isolated, however further studies are needed. Our study indicates produce predominantly carry *E. coli* with a commensal phylogroup and a variety of AMR and virulence-associated traits. Genomic surveillance of bacteria that contaminate produce should be a matter of public health importance in order to develop a holistic understanding of the environmental dimensions of AMR.

## Introduction

The study of antimicrobial resistance (AMR) in food production systems has increased dramatically over the past decade due to the emergence of the One Health framework for understanding AMR and spread of infectious disease in Gram-negative organisms. This is due to the fact that bacteria such as *Escherichia coli* resident in the human gut are typically identified as the causative agent of drug-resistant extra-intestinal infections (Manges and Johnson, 2015). Food is a major contributor to the composition of the human gut microbiota and this environment is also a hotspot of horizontal gene transfer (HGT) where genetic determinants of AMR may be exchanged between commensals and opportunistic pathogens (Lerner et al., 2017). It is therefore important to understand the epidemiology of drug resistant Gram-negative organisms in food so that the relative contribution of food production to drug resistant extraintestinal infections can be determined.

Whilst intensive livestock farming and meat production systems have attracted significant attention (Manges and Johnson, 2015; Manges et al., 2015), produce such as fruit, vegetables and leafy greens must also be considered. Well established as the most common source of intestinal pathogenic *E. coli* infectious outbreaks, such as O157:H7 and O104:H4 in 2011 (EFSA, 2011; Heiman et al., 2015), little is known about the genomic characteristics of commensal *E. coli* in produce, their carriage of AMR determinants, virulence traits and potential presence of opportunistic extraintestinal pathogens. This is surprising considering a number of factors involved in large-scale production of produce.

Firstly, produce is frequently contaminated with *E. coli* from manure-based fertilizers, irrigation water, soil, wild animals and insects (Alegbeleye et al., 2018). Manure and irrigation water are particularly implicated in the direct transfer of *E. coli* to produce, including extended-spectrum beta-lactamase (ESBL)/AmpC producing *E. coli* with high potential for conjugation and HGT (Gao et al., 2015; Njage and Buys, 2015, 2017; Gekenidis et al., 2018a). Furthermore, the presence of antimicrobial residues in manure and sewage sludge used as fertilizer ensures a selective environment for the persistence of drug resistant strains (Li et al., 2013; Rahube et al., 2014; Wolters et al., 2016). Finally, the soil, the putative source of all clinically relevant antimicrobial resistance genes (ARGs) introduces further genetic diversity, uncharacterized antimicrobial compounds and resistance determinants that may drive the emergence of novel resistance and virulence traits in *E. coli* and other *Enterobacteriaceae* (Van Elsas et al., 2003; Djordjevic et al., 2013; Chen et al., 2019). As produce is typically consumed raw, the risk of bacterial survival and transfer to the human gut is likely to be higher than in cooked foods.

Foodborne outbreaks of drug resistant enterohemorrhagic *E. coli* (EHEC) are well documented, and characterized using whole genome sequencing (WGS) (Roy Chowdhury et al., 2015; Gobin et al., 2018). However, genomic data on the full diversity of *E. coli* that colonize produce, their repertoire of ARGs, associated mobile genetic elements (MGEs), virulence-associated genes (VAGs) and the potential presence of extraintestinal pathogenic *E. coli* (ExPEC) is lacking. Just 1% of *E. coli* genomes in Enterobase originate from “Source Type: Plant” and to the best of our knowledge, no publications regarding genomic epidemiological investigations of more than a few genomes exist to date. Molecular studies with limited WGS have identified F, HI2 and N type plasmids in *E. coli* of multiple STs carrying ESBL genes in sprouts and leafy salads in Germany (Freitag et al., 2018), MDR ST1056 *E. coli* in chives (Gekenidis et al., 2018b), a diversity of integrons in multiple Gram-negative bacteria from fruits and vegetables in Portugal (Jones-Dias et al., 2016) and MDR ESBL-producing *E. coli* from irrigation water (Gekenidis et al., 2018a).

Previously, we identified transferable multidrug resistance F and I1 plasmids as well as 63 tetracycline (TET)-resistant *E. coli* carrying F, I1, and HI1 type plasmids in store-bought mixed salad, arugula and cilantro from German supermarkets (Blau et al., 2018). TET-resistant strains were selected due to the extensive use of tetracycline in animal production, which results in transfer of TET-resistant *E. coli* into agricultural ecosystems via manure-based fertilizers (Wolters et al., 2016; Blau et al., 2018). We found that conjugative drug resistance plasmids are associated with the low-abundance or rare microbiota and that were not detected by real time (RT-) PCR in total community DNA without enrichment. Here we performed WGS on an extended collection of 120 TET-resistant *E. coli* strains from the study by Blau et al. (2018) in order to understand their phylogeny, identify STs, ARGs, MGEs, and VAGs.

## Materials and Methods

### Sample Collection, Isolation and DNA Extraction

Sample collection and isolation procedures were previously described (Blau et al., 2018). Briefly, 24 samples of produce purchased from supermarkets in two German cities were analyzed on day 0 and after 7 days of refrigeration. These two groups are hereafter referred to as “fresh” and “stored” samples, respectively. The mixed salad and arugula were purchased from local supermarkets in Braunschweig in June and September 2016, and cilantro was obtained from Asian supermarkets in Braunschweig and Magdeburg in May 2017. An equal number of samples from each produce type were taken. Following Stomacher treatment, TET-resistant *E. coli* were isolated either by direct plating or after enrichment in buffered peptone water at 37°C. *E. coli* were identified on selective media and confirmed by standard biochemical tests. DNA was extracted as previously described and stored at −20°C.

### DNA Sequencing and Assembly

Genomic DNA was prepared for 130 TET-resistant *E. coli* strains using a modified Nextera library preparation protocol and sequenced on an Illumina HiSeq 2500 instrument as previously described (Reid et al., 2017). Raw reads were assembled using Shovill v1.0.4. Assembly statistics were summarized with assembly stats v1.0.1. Ten strains were excluded from the analysis due to incorrect genome sizes.

### Phylogenetic Analysis

SNP-based phylogenetic analysis was performed using Snippy v4.3.6, Gubbins v2.3.4, snp-sites v2.4.1, snp-dists v0.6.3, and FastTree v 2.1.10 as described previously (Reid et al., 2018). The complete genome of *E. coli* K12-MG1655 was used as a reference genome. Alignment of all sequences to the reference genome with Snippy produced a full alignment of 4,720,950 bp. This alignment was then filtered of recombinant regions using Gubbins with default parameters. snp-sites identified conserved variable sites in the Gubbins alignment, producing a final alignment of 16,188 bp. FastTree was then used to generate a maximum-likelihood phylogenetic tree from this alignment. snp-dists was used to calculate pairwise SNP distances on the final alignment between all sequences analyzed. This workflow was also used to generate SNP trees for five of the common STs in the collection. Complete genomes were used as references for ST58 and ST88, whilst Enterobase assemblies were used for the other STs. The reference genomes were as follows ST58 – 90-9281 (gb| CP024243.1); ST88 – 14EC029 (gb| CP024141.1); ST6186 – Enterobase Uberstrain ESC_FA7190AA; ST641 – Enterobase Uberstrain ESC_KA2255AA; ST165 – Enterobase Uberstrain ESC_HA0402AA.

### Gene Screening

Screening for multi-locus STs, ARGs, VAGs, plasmid replicons, e-serotypes and a custom database of additional genes was performed using ARIBA v2.13.3 as previously described (Reid et al., 2018). Phylogroups were determined with ClermonTyper v1.0.0 (Beghain et al., 2018). SNPs in *gyrA* and *parC* genes for fluoroquinolone resistance were identified with NCBI BLAST+ 2.6.0.

### Integron Analysis

BLAST screening was performed with NCBI-BLAST-2.7.1+ to identify scaffolds that carried both *intI1* and ARGs (Camacho et al., 2009). These scaffolds were then annotated in SnapGene v4.1.9 (GSL Biotech) and drawn schematically in Microsoft PowerPoint.

### Data Availability Statement

All short reads and assemblies associated with this study are available at NCBI under BioProject PRJNA563564, individual BioSamples are listed in Supplementary Material. All tree files generated are available at https://github.com/CJREID/WGS_produce_ecoli_2019. SNP distance matrices for individual SNP trees are available in Supplementary Tables S2–S6.

## Results

We performed Illumina WGS on 120 *E. coli* strains from store-bought produce to determine their phylogeny, multi-locus STs, ARGs, class 1 integrons, VAGs and plasmid-associated genes. Strains originated from cilantro (*n* = 102, 85%), arugula (*n* = 15, 12.5%), and mixed salad (*n* = 3, 2.5%).

### Phylogenetic Analysis

Phylogroup B1 dominated the collection, accounting for 70% (*n* = 84) of sequences, followed by A (21%, *n* = 25) and C (4%, *n* = 5). Phylogroups E and F each had two representatives and D one representative. A single strain could not be assigned to any known phylogroup. All 15 *E. coli* strains from arugula belonged to B1, strains from mixed salad belonged to B1 and E, whilst strains from cilantro comprised all phylogroups except E. Phylogroups D, E, and F were only identified in strains isolated after enrichment.

Comparison of isolation method among strains from cilantro showed a decrease in the proportion of B1 and an increase in the proportion of phylogroup A strains from direct plating to enrichment. Twenty-one STs were determined in total, including four single-locus variants and five strains were novel STs. Only two STs were identified in arugula and mixed salad, respectively, whilst 18 STs were present in cilantro as well as the four single-locus variants and novel types.

Common STs included ST6186 (*n* = 37), ST165 (*n* = 17, cilantro only), ST58 (*n* = 14), ST641 (*n* = 8), and ST88 (*n* = 5). Barring ST58, all of these STs were exclusively identified in cilantro. ST6186, ST58, and ST641 belong to phylogroup B1, whilst ST165 and ST88 belong to phylogroup C. Only ST58 and ST224 were isolated from both initial (day 0/fresh) and refrigerated (day 7/stored) samples whereas seven STs were isolated via both direct plating and enrichment methods (Table 1).

**TABLE 1:** Summary of 120 *E. coli* isolated from ready-to-eat cilantro, arugula and mixed salad.

		Direct plating		Enrichment		
Phylogroup	ST	Fresh	Stored	Fresh	Stored	Total
A	*165*				17	17
	*871*			1		1
	*8677*				3	3
	*10-like*	1				1
	*Novel*	3				3
B1	*58*	5		2	7	14
	*212*				1	1
	*224*	1		1	1	3
	*297*				1	1
	*345*			1		1
	*641*	4		4		8
	*1250*			1		1
	*1704*	1				1
	*1727*	2				2
	*2165*	1		1		2
	*4684*		3		1	4
	*5891*				3	3
	*6021*	1		1		2
	*6186*		15		22	37
	*124-like*			1		1
	*165-like*				1	1
	*6186-like*				1	1
	*Novel*	1				1
C	*88*	2		3		5
D	*69*				1	1
E	*7576*			2		2
F	*457*				2	2
Unknown	*Novel*			1		1
Total		22	18	19	61	120

*Green cells highlight arugula samples and purple cells highlight mixed salad samples. All other samples originated from cilantro.*

**FIGURE 1:**
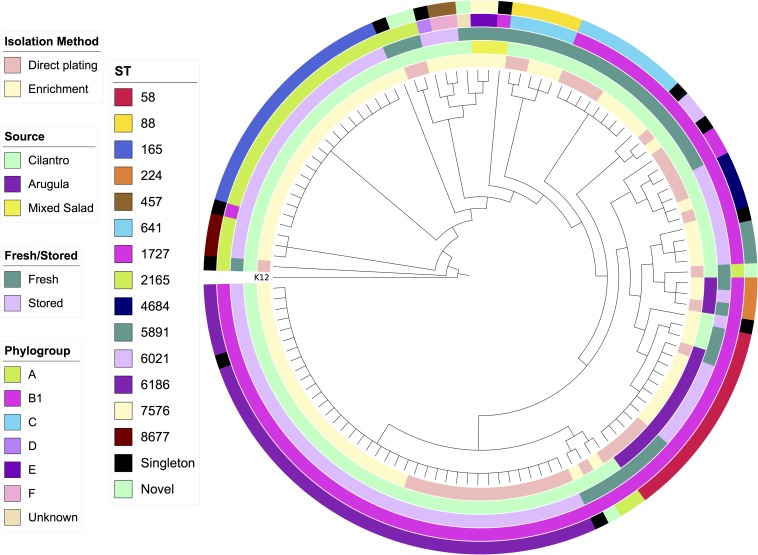
Maximum-likelihood SNP-based cladogram of 120 *E. coli* from produce based on reference genome *E. coli* K12-MG1655. Metadata rings labeled from innermost to outermost; isolation method, source, fresh/stored, phylogroup, Achtman ST.

SNP-based core genome phylogeny based on K12-MG1655 clearly stratified STs, however, there were some anomalous results with respect to phylogroup, such as an A strain in the B1 clade and a B1 strain in the A clade (Figure 1). This may be due to truncations or SNPs in the primer binding sites that ClermonTyper screens for resulting in misidentification.

In addition to the SNP tree encompassing all strains, we generated SNP-based core genome phylogenies for the most common STs to determine whether they were diverse or clonal. We utilized publicly available sequences of the same ST as references, and complete genomes where possible. ST6186, ST165, and ST641 strains were near identical at a core genome level with sequences only separated by 0–3 SNPs (Supplementary Tables S2–S4). Despite this, mapping accessory genomic elements to the full SNP tree indicates that genomic variability still exists among these highly clonal strains, a phenomenon recently identified in a gastrointestinal population of ST131 (Forde et al., 2019). By contrast, ST58 and ST88 strains each consisted of two separate lineages (Supplementary Tables S5, S6). Twelve ST58 strains from arugula were separated by only 0–2 SNPs whilst the second lineage comprised two strains from cilantro separated from the first lineage by 402 and 403 SNPs, respectively. These two strains carried *bla*_CTX–M–__15_ ESBL genes. Similarly, ST88 comprised three near identical strains (0–1 SNPs) and two strains belonging to another lineage, separated from the first by 316 and 317 SNPs, respectively. All five of these strains were isolated from the fresh cilantro samples, however the first three were from enrichment culture, whilst the latter two were from direct plating. In both ST58 and ST88, separate lineages exhibited distinct accessory gene contents. For example, the two divergent ST58 strains carried a larger array of ARGs including *bla*_CTX–M–__15_ in contrast to the other 12, which only carried a multi-drug efflux pump gene *mdfA* and *tet*(B). The ST88 strains all exhibited extensive VAG arrays particularly with regard to genes involved in iron acquisition, however the three strains from the first lineage carried ColV-associated genes *cvaABC*, *cvi*, and *cma* whereas the second lineage did not.

### Virulence-Associated Genes

**FIGURE 2:**
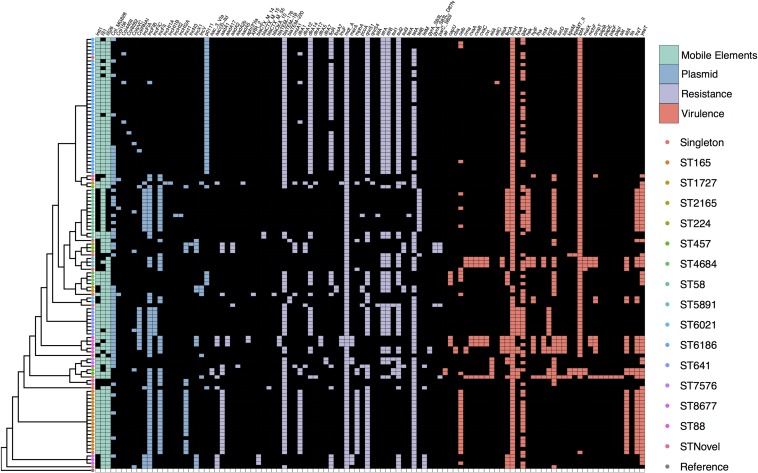
SNP cladogram from Figure 1 mapped against gene presence and absence including fluoroquinolone resistance SNPs. Colored tree nodes indicate strain ST. Colored squares indicate presence of genes: green - mobile elements; blue - plasmid replicons; purple - resistance genes; red - virulence-associated genes.

We screened the collection for VAGs reported in a variety of *E. coli* pathotypes and found that overall virulence carriage was relatively low with an average of six VAGs per strain. The abundant ST6186 sequences typically only carried *fimH* and *lpfA*. The most common virulence genes were the widespread fimbrial adhesin gene *fimH* (96%, *n* = 115), long polar fimbriae gene *lpfA* (78%, *n* = 93) and glutamate decarboxylase gene *gad* (63%, *n* = 76). It was apparent that VAG carriage patterns generally corresponded with specific STs and a small subset of STs demonstrated an extensive array of VAGs (Figure 2). Notable among these were three ST5891, three ST88, and one ST69 strain, all of which carried greater than 15 VAGs as well as genetic indicators of ColV plasmid carriage including *cvaABC*, *cma*, *cvi*, *iroN*, and *iss*. Capsular polysaccharide gene *kpsM*, associated with ExPEC infection was present in three phylogroup D strains, two of these were ST457 and one was ST69. Shiga-toxin genes and genes associated with EHEC were not detected.

### Antimicrobial Resistance and Plasmid Replicon Genes

We identified 42 ARGs in the collection with each strain carrying an average of seven. All 120 strains carried the multidrug efflux pump *mdfA.* The most common genes known to confer resistance to a specific class of antimicrobial included *tet*(A) (78%, *n* = 93), *bla*_TEM–__1__B_ (74%, *n* = 89), *qnrS1* (70%, *n* = 84), *sul2* (60%, *n* = 72), *strA-strB* (54%, *n* = 65), and *dfrA14* (48%, *n* = 57). ESBL genes were present albeit in low numbers. Two ST58 strains carried *bla*_CTX–M–__15_, three ST8677 (CC10) strains carried *bla*_CTX–M–__14_, one ST871 strain carried *bla*_CTX–M–__55_, and a single ST1704 strain carried *bla*_OXA–__10_. SNPs in *gyrA* and *parC* that confer high level resistance to fluoroquinolones were present in three ST224 strains and one ST345 strain. Despite the fact that strains were selected for phenotypic resistance to tetracycline, 14 strains did not carry a known tetracycline (*tet*) resistance gene variant. Similar to the virulence profiles, sub-clades of the SNP tree with more than one representative typically displayed the same ARGs, though there were examples of variability (Figure 2).

The most common plasmid replicons observed were FIB (55%, *n* = 66), Col-MG828 (74%, *n* = 55), FII (43%, *n* = 51), pO111 (37%, *n* = 44), and I1 (19%, *n* = 23). The pO111 replicon was present in all ST6186 and ST4684 strains, these STs also carried near identical suites of ARGs. The combination of I1, FII, and FIB was present in all ST165 strains bar one that lacked an I1 replicon. An increase in the abundance of pO111, FII, and I1 replicons from fresh to stored samples corresponded to with the emergence of ST6186 and ST165 strains that carried them. All seven strains carrying ColV determinants carried F type replicons.

### Class 1 Integrons

**FIGURE 3:**
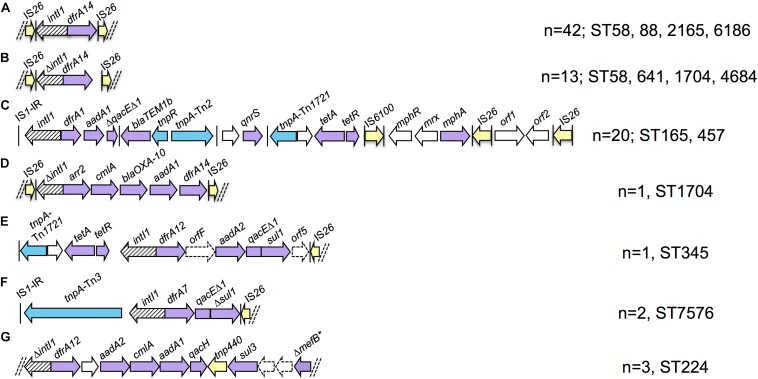
Schematic representation of integron structures identified in draft assemblies (not to scale). Purple arrows - resistance genes; yellow arrows - insertion sequences; blue arrows – transposon-associated genes. Vertical bars indicate inverted repeats. Parallel dashed bars indicate scaffold breaks. Letters **(A–G)** refer to the different integron types identified and referred to in text.

Eighty-four strains were positive for *intI1* and carried eight ARGs on average compared to an average of four ARGs for *intI1* negative strains. We performed *de novo* assemblies in order to characterize the context of resistance genes associated with class 1 integrons. Almost all integron scaffolds were flanked by IS elements, predominantly partial copies of IS*26* and IS*1* truncated due to scaffold breaks. Seven unique structures were identified on complete scaffolds (Figure 3) and alignment of short reads to these structures identified other strains that carried the same structures. Integron type A was present in 42 strains of four different STs (ST58, ST88, ST2165, and ST6186/ST6186-like) and consisted of *intI1* and a *dfrA14* gene cassette. Partial copies of IS*26* flanked the truncated integron with only 6 bp upstream of *intI1* and 7 bp downstream of *dfrA14* remaining, respectively. Integron type B, present in 13 strains of four STs (ST58, ST641, ST1704, and ST4684), was nearly identical, however the initial IS*26* deleted the final 8 bp of *intI1*, whilst the terminal IS*26* was further downstream of *dfrA14* leaving 183 bp of the 3′-*CS* remaining. Integron type C was a highly mosaic arrangement observed in 20 strains of two STs [ST165/ST165-like (*n* = 18) and ST457 (*n* = 2)]. An inverted repeat of IS*1* was present upstream of *intI1*, *dfrA1-aadA1* gene cassettes and 283 bp of *qacEΔ1*, which was truncated by a complete copy of Tn*2*. A hypothetical protein, *qnrS1* and an incomplete copy of Tn*1721* containing *tet*(A)-*tet*(R) followed, preceding IS*6100*, *mphR-mrx-mphA* macrolide resistance genes and an IS*26* flanked module containing genes encoding a DNA cytosine methyltransferase and *Eco*RII restriction enzyme. Integron type D was only present in one ST1704 strain and consisted of IS*26-ΔintI1-arr2-cmlA-bla*_OXA–__10_-*aadA1-dfrA14-*IS*26*. The terminal IS*26* insertion was identical to integron type B residing 183 bp downstream of *dfrA14*. Integron type E was similarly only present in one ST345 strain. This arrangement comprised a partial copy of Tn*1721* followed by *intI1-dfrA12-orfF-aadA2-qacEΔ1-sul1-orf5-tniB-ΔtniA-*IS*26*. Upstream of this resistance region were a copper/silver resistance operon and numerous hypothetical proteins similar to those described in HI2 plasmids from Australia and China (Fang et al., 2016; Billman-Jacobe et al., 2018; Wyrsch et al., 2019). Integron type F was present in two ST7576 strains and consisted of an IS*1* inverted repeat, a Tn*3-*like transposase gene *tnpA*, a recombinase family protein gene then *intI1-dfrA7-qacEΔ1-Δsul1-*IS*26*. Integron type G was a *sul3* type integron present in three ST224 strains. No IS elements were present at either end of the scaffold, however genes at either end were truncated. The structure comprised *ΔintI1-dfrA12-orfF-aadA2-cmlA1-aadA1-qacH-tnp440-sul3-orfAB-ΔmefB*.

## Discussion

### Phylogroup B1 and a Selection of Sequence Types Dominate Produce

This study identified numerous phylogenomic characteristics of *E. coli* from German store-bought cilantro, arugula and mixed salad. Due to the strong bias toward cilantro in our collection it is not appropriate to make direct comparisons between the findings for each produce type, however a number of broader conclusions can be reached. Firstly, the number of isolates obtained from cilantro indicated they were more contaminated with TET-resistant *E. coli* than arugula or mixed salad samples. This may reflect production practices, including but not limited to hygiene and antimicrobial use, that are specific to the producer or country of origin. Conversely, it may indicate that *E. coli* are simply better adapted to colonizing cilantro as opposed to arugula and mixed salad. Regardless, it is necessary to explore this in future studies so that risk factors for *E. coli* contamination of produce can be identified.

With regard to phylogeny, the dominance of phylogroup B1 was notable. Phylogroup B1 is typically considered to be an environmental lineage, however human extra-intestinal infections, including sepsis caused by B1 *E. coli* such as ST58 are reported (McKinnon et al., 2018). It is difficult to compare to other studies that sample different types of produce including leafy greens and vegetables, however phylogroup B1 similarly dominated 68 samples of *E. coli* from farmers’ market lettuce in British Columbia (Wood et al., 2015). Contrastingly, studies on cabbage and spinach in South Africa and vegetables in Mexico both determined phylogroup A to be most prevalent (Plessis et al., 2017; Corzo-Ariyama et al., 2019). A Portuguese study found a fairly even distribution between B1, A and D in ready-to-eat salads (Campos et al., 2013). In another Portuguese study, phylogroups A and B1 were the most prevalent both in irrigation water and vegetables (Araujo et al., 2017). This suggests that phylogroups of *E. coli* from produce exhibit distributions that differ geographically and are influenced by the type of produce sampled.

We identified 21 known STs in the collection, four single-locus variants and five strains of novel types. The dominance of ST6186 in cilantro was intriguing. Firstly, all 37 strains belonging to this ST were *intI1* positive, genetically multidrug resistant and highly clonal at a core genome level. However, it was clear that they possessed variable accessory genomes. This suggests that ST6186 *E. coli* may possess core genetic characteristics that enable it to successfully colonize cilantro and that accessory gene gain and loss occurs within the plant microenvironment. It is a concern that this dominant ST also carries class 1 integrons and multiple ARGs that could be transferred to humans by consumption of produce. ST6186 is yet to be reported in any publications and only four examples exist in Enterobase as of May 2019, therefore it is not possible to comment on the origin of this ST in our collection. The second most common ST165 has been reported in Portuguese street food and from the feces of a healthy human in Tunisia (Ben Sallem et al., 2012; Campos et al., 2015). ST641 has been identified in diseased horses in France, Sweden and the Netherlands carrying *bla*_CTX–M–__1_ in association with IncHI1 plasmids (Apostolakos et al., 2017; Lupo et al., 2018), in the Colombian poultry chain harboring ESBL/AmpC genes on IncI1 plasmids (Castellanos et al., 2017), and in ESBL producing *E. coli* in irrigation water and German biogas plants (Schauss et al., 2015; Gekenidis et al., 2018a). We also identified a single ST69, which is a known global pandemic ExPEC strain commonly associated with poultry (Riley, 2014).

Two common STs of particular interest were ST58 and ST88. ST58 has emerged worldwide in wild and food-production animals, the environment as well as human infections (Chah et al., 2018; Gekenidis et al., 2018a; McKinnon et al., 2018; Sacramento et al., 2018; Irenge et al., 2019; Zurfluh et al., 2019). Carriage of ARGs conferring resistance to critically important third-generation cephalosporins and colistin were noted in these reports. ESBL positive ST58 have been isolated in Germany from cattle, poultry, biogas plants, and human infections (Schauss et al., 2015; Hammerl et al., 2018; Pietsch et al., 2018). Two of our fourteen ST58 strains carried ESBL gene *bla*_CTX–M–__15_, however different ESBL genes were identified in the aforementioned studies.

Five ST88 strains from cilantro were present in our collection. Three of these were phylogenetically distinct from the other two and carried genetic indicators of ColV plasmid carriage as well as extensive VAG arrays. A study from Brazil identified an ST88 strain from a human extra-intestinal infection that also exhibited the hallmarks of ColV carriage suggesting those in our collection may be ExPEC (Maluta et al., 2014). ST88 has a global range in humans and food production animals (Day et al., 2016; Yamaji et al., 2018). It is frequently identified carrying CTX-M family ESBLs and may exhibit a variety of pathotypes including ETEC and STEC indicating significant genetic and phenotypic variability (Bai et al., 2016; Kusumoto et al., 2016; Dierikx et al., 2018). A study of ESBL positive *E. coli* from a Swiss ready-to-eat salad production facility identified ST88 in irrigation water (Nüesch-Inderbinen et al., 2015). ST58 and ST88 strains both exhibited separate lineages at the core genome level, as well as variable ARG and VAG carriage, indicating that the presence of each ST is probably attributable to multiple sources. This reflects their wide epidemiological range and known heterogeneity. Further SNP-based phylogenomic studies of each ST, comprising strains from a variety of sources are necessary to assess potential origins of the produce strains.

Whilst our sample size is too small to draw any firm conclusions, it seems likely that storage has an effect on the abundance of different lineages. This was seen in the emergence of ST6186 and ST165 and the plasmid types they carried in stored samples and may be relevant to improving food safety in the future.

### Successful Sequence Types Are Responsible for the Majority of AMR Genotypes

Patterns of plasmid replicon and ARG carriage strongly matched the SNP phylogeny (Figure 2) indicating that certain STs were primarily responsible for the abundance of AMR in the collection. This was also evident when comparing fresh to stored samples as plasmid replicon presence changed with ST abundance. In contrast, integron types A, B, and C were present in multiple STs indicating the occurrence of HGT between *E. coli* in produce or exposure to a common source of ARGs prior to arrival in produce. A combination of both is most likely. Further long read sequencing of strains with similar plasmid and ARG carriage is necessary to assess this. The absence of *tet* genes from some strains was notable and suggests yet uncharacterized *tet* genes are carried by these strains.

### Class 1 Integrons Are Associated With IS26 and Have Diverse Origins

While the limitations of short-read WGS can make it difficult to characterize resistance regions, we were able to identify a number of integrons present in the collection. A key feature of the integrons in this collection was the presence of atypical integrons. Four of seven structures lacked a complete 3′-*CS* and IS*26-*like inverted repeats were often present at the ends of scaffolds. Partial copies of direct facing IS*26* flanked three integron scaffolds, including one carrying *bla*_OXA–__10_. The association of IS*26* with integrons and ARGs is a multi-faceted issue due to the unique activities of IS*26* relative to other IS elements. IS*26* flanked integrons and ARGs may be mobilized as transposable units (TUs), incorporate into the chromosome, virulence plasmids and target existing copies of IS*26* on other DNA molecules (Roy Chowdhury et al., 2015, 2018; Harmer and Hall, 2016; Moran and Hall, 2018; Oliva et al., 2018). This is a great concern as it confers increased mobility to ARGs, enables antibiotic co-selection of virulence traits and drives further evolution of multidrug resistance regions. One benefit of IS*26* insertions in resistance regions is the diagnostic genetic signatures they generate. Some truncations are globally disseminated whilst others have a more specific distribution and with relevant epidemiological data may facilitate inference of the origins and movements of certain plasmids and mobile ARGs.

Integron types A and B were similar, differing by the sites of IS*26* insertions. Integron type A had no exact matches in the NCBI Nucleotide database, however, integron type B was present in more than 100 entries, comprising multiple species of *Enterobacteriaceae* from multiple sources. Sections of integron type C were previously identified, however, the entire modular structure was unique. The IS*26* flanked integron type D containing *bla*_OXA–__10_ was identical to three unpublished Chinese *E. coli* plasmid sequences isolated from poultry, river water and an unknown source (gb| CP033636.1, gb| CP010168.1, gb| KY421937.1). Integron type E was previously described in three MDR *E. coli* on HI2 plasmids in Australian swine (Billman-Jacobe et al., 2018; Wyrsch et al., 2019). Integron type F was identical to that described on the chromosome of multiple O104:H4 *E. coli* responsible for the 2011 produce-associated outbreak in Germany (Roy Chowdhury et al., 2015). This indicates the plasmids or mobile elements that carry this integron may have contributed to their dissemination to other lineages of *E. coli*. The scaffold containing integron type G broke at both ends without the presence of IS elements, however read-mapping with ARIBA indicated that the truncation of *mefB* left 260 bp of the macrolide efflux gene remaining, an arrangement that is globally disseminated in swine, poultry and wild animals (Alonso et al., 2016, 2017; Reid et al., 2017). Altogether our data indicate that integrons present in produce come from multiple sources, though food-production animals may be particularly implicated. Far more genome sequences of *E. coli* from produce, food-production animals and associated sources of irrigation water are required to explore this properly. The presence of previously unidentified integrons in multiple STs suggests that evolution and HGT of drug resistance loci occurs in *E. coli* in produce.

### Virulence Genes Are Not Common, However, the Presence of ColV Is Concerning

Virulence-associated gene carriage was low overall and VAGs associated with enteropathogenic *E. coli* subtypes were not detected. This is consistent with the sporadic nature of such contamination events that lead to outbreaks and suggests that produce are not typically colonized by these pathotypes. Furthermore, the diversity of VAG patterns between strains suggests that VAG carriage is unrelated to successful colonization in this niche. With respect to ExPEC VAGs, the presence of genes indicating ColV plasmid carriage in ST88, ST5891, and ST69 strains from cilantro is a concern as these plasmids are implicated in extra-intestinal pathogenicity in humans and poultry and have been identified carrying multiple ARGs, including *tet* genes (Moran and Hall, 2018). This may be indicative of poultry-associated *E. coli* as a contaminating source or human contamination during harvest and packaging. Sources of irrigation water, fertilizer and details on human handling of these products could be investigated to determine the origin of these strains. Co-carriage of multiple ARGs in these strains allows antibiotic selection of ColV plasmids and may lead to increased incidence of ExPEC in produce. As such, produce should be monitored for carriage of hybrid virulence-resistance plasmids. Long-read sequencing of these strains to fully characterize ColV plasmids and any ARGs they may carry will be performed in order to contribute to this.

### Methodology and Limitations

The methodology used in this study raises a number of important considerations for future genomic epidemiological studies of produce. It should be noted that this study was not explicitly designed to compare sampling methods directly. Indeed, the relative abundance of isolates from cilantro confounded a clear comparison between sampling methods for each produce type. It is important that future studies control for imported or local status and potentially eschew selection based on AMR phenotypes in order to accurately estimate the rates of *E. coli* contamination in produce as well as the abundance of ARGs and MGEs.

Despite this limitation, it is apparent from this study and previous work that enrichment prior to plating has some effect on both STs and the mobile resistome that may be observed (Blau et al., 2018). It is important to understand what is present even at low abundance in produce as rare strains and plasmids carrying ARGs may carry a fitness advantage and expand in altered conditions post-purchase such as refrigeration and in the human gastrointestinal tract after consumption. Indeed, it seems likely that periods of refrigeration alter the composition of *E. coli* in produce, as seen by the emergence of a number of STs in refrigerated samples that were not isolated from fresh samples. The repeated isolation of MDR ST6186 from the refrigerated samples by both direct plating and enrichment is an interesting example of this. It is probable that some lineages of *E. coli* have greater tolerance to, and ability to grow during periods of low temperature. This is important, particularly in the context of AMR, as the time between purchase and consumption could have a significant effect on the lineages and AMR characteristics of *E. coli* that reach and colonize the human gastrointestinal tract.

The selection of TET-resistant strains obviously introduces a bias to the *E. coli* populations being examined, however we believe this is justified given the aim of characterizing the repertoire of AMR genes and associated MGEs in produce that may reach the human gut via consumption. The extensive use of tetracycline in animal husbandry and its known contamination of agro-ecosystems informed the specific selection of TET-resistant strains. We acknowledge that developing a full ecological appreciation of *E. coli* in produce and the changes that occur during refrigeration requires an unbiased approach that may be pursued in the future.

Short-read sequencing is evidently a step forward from classical molecular techniques and very good for identifying the different *E. coli* lineages, the ARGs and VAGs they carry. However, long-read sequencing of AMR plasmids from produce is required to localize specific genes to plasmids, and plasmids to lineages of *E. coli* and other *Enterobacteriaceae*. Large databases of long-read plasmid sequences from diverse sources are required to truly understand their epidemiology and assess the role of produce in the complex web of interactions that underpin the global issue of AMR.

## Conclusion

To the best of our knowledge this is the first WGS study of a collection of *E. coli* from produce. We identified (i) a diversity of multi-locus STs, some of which were previously reported to be associated with food animals, irrigation water and human disease; (ii) a wide variety of ARGs and class 1 integrons associated with IS*26* and (iii) VAGs indicating the presence of ColV plasmids. This data and known factors involved in the production of produce indicate that food production animals, manure- and sewage sludge-based fertilizer, irrigation water and its sources should be further investigated with WGS. This could allow accurate determination of the origins of *E. coli* that contaminate produce and monitor them over time to inform AMR mitigation measures across the spectrum of agriculture. Furthermore, having established the need for isolation in order to characterize the rare microbiota, it is necessary to undertake further studies to understand the dynamics of certain lineages of epidemiologically relevant organisms and elucidate the relative contribution of clonal dissemination and HGT to the emergence of AMR in produce.

## Data Availability Statement

The datasets generated for this study can be found in the NCBI Bioproject PRJNA563564, https://github.com/CJREID/WGS_produce_ecoli_2019.

## Author Contributions

CR was responsible for investigation, data curation, formal analysis, software, methodology, validation, visualization, writing the original draft, review, and editing. KB was responsible for preliminary investigation, data curation, formal analysis, review, and editing. SJ was responsible for conceptualization, resources, data curation, and investigation. KS and SD were responsible for conceptualization, funding acquisition, supervision, review, and editing.

## Conflict of Interest

The authors declare that the research was conducted in the absence of any commercial or financial relationships that could be construed as a potential conflict of interest.

## References

[B1] Alegbeleye O. O. Singleton I. Sant’Ana A. S. (2018). Sources and contamination routes of microbial pathogens to fresh produce during field cultivation: a review. *Food Microbiol.* 73 177–208. 10.1016/j.fm.2018.01.003 29526204 PMC7127387

[B2] Alonso C. A. Gonzalez-Barrio D. Tenorio C. Ruiz-Fons F. Torres C. (2016). Antimicrobial resistance in faecal *Escherichia coli* isolates from farmed red deer and wild small mammals. Detection of a multiresistant *E. coli* producing extended-spectrum beta-lactamase. *Comp. Immunol. Microbiol. Infect. Dis.* 45 34–39. 10.1016/j.cimid.2016.02.003 27012919

[B3] Alonso C. A. Michael G. B. Li J. Somalo S. Simon C. Wang Y. (2017). Analysis of blaSHV-12-carrying *Escherichia coli* clones and plasmids from human, animal and food sources. *J. Antimicrob. Chemother.* 72 1589–1596. 10.1093/jac/dkx024 28333184

[B4] Apostolakos I. Franz E. van Hoek A. Florijn A. Veenman C. Sloet-van Oldruitenborgh-Oosterbaan M. M. (2017). Occurrence and molecular characteristics of ESBL/AmpC-producing *Escherichia coli* in faecal samples from horses in an equine clinic. *J. Antimicrob. Chemother.* 72 1915–1921. 10.1093/jac/dkx072 28333298

[B5] Araujo S. I Silva A. T. Tacao M. Patinha C. Alves A. Henriques I. (2017). Characterization of antibiotic resistant and pathogenic *Escherichia coli* in irrigation water and vegetables in household farms. *Int. J. Food Microbiol.* 257 192–200. 10.1016/j.ijfoodmicro.2017.06.020 28668729

[B6] Bai L. Hurley D. Li J. Meng Q. Wang J. Fanning S. (2016). Characterisation of multidrug-resistant Shiga toxin-producing *Escherichia coli* cultured from pigs in China: co-occurrence of extended-spectrum beta-lactamase- and mcr-1-encoding genes on plasmids. *Int. J. Antimicrob. Agents* 48 445–448. 10.1016/j.ijantimicag.2016.06.021 27526978

[B7] Beghain J. Bridier-Nahmias A. Le Nagard H. Denamur E. Clermont O. (2018). ClermonTyping: an easy-to-use and accurate in silico method for *Escherichia* genus strain phylotyping. *Microb. Genom.* 4:e000192. 10.1099/mgen.0.000192 29916797 PMC6113867

[B8] Ben Sallem R. Ben Slama K. Estepa V. Jouini A. Gharsa H. Klibi N. (2012). Prevalence and characterisation of extended-spectrum beta-lactamase (ESBL)-producing *Escherichia coli* isolates in healthy volunteers in Tunisia. *Eur. J. Clin. Microbiol. Infect. Dis.* 31 1511–1516. 10.1007/s10096-011-1471-z 22065280

[B9] Billman-Jacobe H. Liu Y. Haites R. Weaver T. Robinson L. Marenda M. (2018). pSTM6-275, a conjugative IncHI2 plasmid of *Salmonella enterica* that confers antibiotic and heavy-metal resistance under changing physiological conditions. *Antimicrob. Agents Chemother.* 62:e02357-17. 10.1128/aac.02357-17 29439975 PMC5923156

[B10] Blau K. Bettermann A. Jechalke S. Fornefeld E. Vanrobaeys Y. Stalder T. (2018). The transferable resistome of produce. *mBio* 9:e01300-18. 10.1128/mBio.01300-18 30401772 PMC6222124

[B11] Camacho C. Coulouris G. Avagyan V. Ma N. Papadopoulos J. Bealer K. (2009). BLAST+: architecture and applications. *BMC Bioinformatics* 10:421. 10.1186/1471-2105-10-421 20003500 PMC2803857

[B12] Campos J. Gil J. Mourao J. Peixe L. Antunes P. (2015). Ready-to-eat street-vended food as a potential vehicle of bacterial pathogens and antimicrobial resistance: an exploratory study in Porto region, Portugal. *Int. J. Food Microbiol.* 206 1–6. 10.1016/j.ijfoodmicro.2015.04.016 25910073

[B13] Campos J. Mourao J. Pestana N. Peixe L. Novais C. Antunes P. (2013). Microbiological quality of ready-to-eat salads: an underestimated vehicle of bacteria and clinically relevant antibiotic resistance genes. *Int. J. Food Microbiol.* 166 464–470. 10.1016/j.ijfoodmicro.2013.08.005 24036261

[B14] Castellanos L. R. Donado-Godoy P. Leon M. Clavijo V. Arevalo A. Bernal J. F. (2017). High heterogeneity of *Escherichia coli* sequence types harbouring ESBL/AmpC genes on IncI1 plasmids in the Colombian poultry chain. *PLoS One* 12:e0170777. 10.1371/journal.pone.0170777 28125687 PMC5268450

[B15] Chah K. F. Ugwu I. C. Okpala A. Adamu K. Y. Alonso C. A. Ceballos S. (2018). Detection and molecular characterisation of extended-spectrum beta-lactamase-producing enteric bacteria from pigs and chickens in Nsukka, Nigeria. *J. Glob. Antimicrob. Resist.* 15 36–40. 10.1016/j.jgar.2018.06.002 29908916

[B16] Chen Q.-L. Cui H.-L. Su J.-Q. Penuelas J. Zhu Y.-G. (2019). Antibiotic resistomes in plant microbiomes. *Trends Plant Sci.* 24 530–541. 10.1016/j.tplants.2019.02.010 30890301

[B17] Corzo-Ariyama H. A. Garcia-Heredia A. Heredia N. Garcia S. Leon J. Jaykus L. (2019). Phylogroups, pathotypes, biofilm formation and antimicrobial resistance of *Escherichia coli* isolates in farms and packing facilities of tomato, jalapeno pepper and cantaloupe from Northern Mexico. *Int. J. Food Microbiol.* 290 96–104. 10.1016/j.ijfoodmicro.2018.10.006 30317111

[B18] Day M. J. Rodriguez I. van Essen-Zandbergen A. Dierikx C. Kadlec K. Schink A. K. (2016). Diversity of STs, plasmids and ESBL genes among *Escherichia coli* from humans, animals and food in Germany, the Netherlands and the UK. *J. Antimicrob. Chemother.* 71 1178–1182. 10.1093/jac/dkv485 26803720

[B19] Dierikx C. M. van der Goot J. van Essen-Zandbergen A. Mevius D. J. (2018). Dynamics of cefotaxime resistant *Escherichia coli* in broilers in the first week of life. *Vet. Microbiol.* 222 64–68. 10.1016/j.vetmic.2018.07.001 30080674

[B20] Djordjevic S. P. Stokes H. W. Roy Chowdhury P. (2013). Mobile elements, zoonotic pathogens and commensal bacteria: conduits for the delivery of resistance genes into humans, production animals and soil microbiota. *Front. Microbiol.* 4:86. 10.3389/fmicb.2013.00086 23641238 PMC3639385

[B21] EFSA (2011). Shiga toxin-producing *E. coli* (STEC) O104:H4 2011 outbreaks in Europe: taking stock. *EFSA J.* 9:2390 10.2903/j.efsa.2011.2390

[B22] Fang L. Li X. Li L. Li S. Liao X. Sun J. (2016). Co-spread of metal and antibiotic resistance within ST3-IncHI2 plasmids from *E. coli* isolates of food-producing animals. *Sci. Rep.* 6:25312. 10.1038/srep25312 27143648 PMC4855149

[B23] Forde B. M. Roberts L. W. Phan M. D. Peters K. M. Fleming B. A. Russell C. W. (2019). Population dynamics of an *Escherichia coli* ST131 lineage during recurrent urinary tract infection. *Nat. Commun.* 10:3643. 10.1038/s41467-019-11571-5 31409795 PMC6692316

[B24] Freitag C. Michael G. B. Li J. Kadlec K. Wang Y. Hassel M. (2018). Occurrence and characterisation of ESBL-encoding plasmids among *Escherichia coli* isolates from fresh vegetables. *Vet. Microbiol.* 219 63–69. 10.1016/j.vetmic.2018.03.028 29778206

[B25] Gao L. Hu J. Zhang X. Wei L. Li S. Miao Z. (2015). Application of swine manure on agricultural fields contributes to extended-spectrum β-lactamase-producing *Escherichia coli* spread in Tai’an, China. *Front. Microbiol.* 6:313 10.3389/fmicb.2015.00313 PMC4396445 25926828

[B26] Gekenidis M. T. Qi W. Hummerjohann J. Zbinden R. Walsh F. Drissner D. (2018a). Antibiotic-resistant indicator bacteria in irrigation water: high prevalence of extended-spectrum beta-lactamase (ESBL)-producing *Escherichia coli*. *PLoS One* 13:e0207857. 10.1371/journal.pone.0207857 30475879 PMC6258136

[B27] Gekenidis M. T. Schoner U. von Ah U. Schmelcher M. Walsh F. Drissner D. (2018b). Tracing back multidrug-resistant bacteria in fresh herb production: from chive to source through the irrigation water chain. *FEMS Microbiol. Ecol.* 94:fiy149. 10.1093/femsec/fiy149 30101286 PMC6138756

[B28] Gobin M. Hawker J. Cleary P. Inns T. Gardiner D. Mikhail A. (2018). National outbreak of Shiga toxin-producing *Escherichia coli* O157:H7 linked to mixed salad leaves, United Kingdom, 2016. *Euro Surveill.* 23:17-00197. 10.2807/1560-7917.ES.2018.23.18.17-00197 29741151 PMC6053625

[B29] Hammerl J. A. Irrgang A. Grobbel M. Tenhagen B. A. Kasbohrer A. (2018). Complete genome sequence of a blaCTX-M-1-Harboring *Escherichia coli* isolate recovered from cattle in Germany. *Genome Announc.* 6:e01476-17. 10.1128/genomeA.01476-17 29371351 PMC5786677

[B30] Harmer C. J. Hall R. M. (2016). IS26-mediated formation of transposons carrying antibiotic resistance genes. *mSphere* 1:e00038-16. 10.1128/mSphere.00038-16 27303727 PMC4894685

[B31] Heiman K. E. Mody R. K. Johnson S. D. Griffin P. M. Gould L. H. (2015). *Escherichia coli* O157 outbreaks in the United States, 2003-2012. *Emerg. Infect. Dis.* 21 1293–1301. 10.3201/eid2108.141364 26197993 PMC4517704

[B32] Irenge L. M. Ambroise J. Bearzatto B. Durant J. F. Chirimwami R. B. Gala J. L. (2019). Whole-genome sequences of multidrug-resistant *Escherichia coli* in South-Kivu Province, Democratic Republic of Congo: characterization of phylogenomic changes, virulence and resistance genes. *BMC Infect. Dis.* 19:137. 10.1186/s12879-019-3763-3 30744567 PMC6371417

[B33] Jones-Dias D. Manageiro V. Ferreira E. Barreiro P. Vieira L. Moura I. B. (2016). Architecture of Class 1, 2, and 3 integrons from gram negative bacteria recovered among fruits and vegetables. *Front. Microbiol.* 7:1400. 10.3389/fmicb.2016.01400 27679611 PMC5020092

[B34] Kusumoto M. Hikoda Y. Fujii Y. Murata M. Miyoshi H. Ogura Y. (2016). Emergence of a multidrug-resistant shiga toxin-producing enterotoxigenic *Escherichia coli* lineage in diseased swine in Japan. *J. Clin. Microbiol.* 54 1074–1081. 10.1128/jcm.03141-15 26865687 PMC4809958

[B35] Lerner A. Matthias T. Aminov R. (2017). Potential effects of horizontal gene exchange in the human gut. *Front. Immunol.* 8:1630. 10.3389/fimmu.2017.01630 29230215 PMC5711824

[B36] Li Y. X. Zhang X. L. Li W. Lu X. F. Liu B. Wang J. (2013). The residues and environmental risks of multiple veterinary antibiotics in animal faeces. *Environ. Monit. Assess.* 185 2211–2220. 10.1007/s10661-012-2702-1 22692716

[B37] Lupo A. Haenni M. Saras E. Gradin J. Madec J. Y. Borjesson S. (2018). Is blaCTX-M-1 riding the same plasmid among horses in Sweden and France? *Microb. Drug Resist*. 10.1089/mdr.2017.0412 [Epub ahead of print]. 29792781

[B38] Maluta R. P. Logue C. M. Casas M. R. Meng T. Guastalli E. A. Rojas T. C. (2014). Overlapped sequence types (STs) and serogroups of avian pathogenic (APEC) and human extra-intestinal pathogenic (ExPEC) *Escherichia coli* isolated in Brazil. *PLoS One* 9:e105016. 10.1371/journal.pone.0105016 25115913 PMC4130637

[B39] Manges A. R. Harel J. Masson L. Edens T. J. Portt A. Reid-Smith R. J. (2015). Multilocus sequence typing and virulence gene profiles associated with *Escherichia coli* from human and animal sources. *Foodborne Pathog. Dis.* 12 302–310. 10.1089/fpd.2014.1860 25774654

[B40] Manges A. R. Johnson J. R. (2015). Reservoirs of extraintestinal pathogenic *Escherichia coli*. *Microbiol. Spectr.* 3 1–12. 10.1128/microbiolspec.UTI-0006-2012 26542041

[B41] McKinnon J. Roy Chowdhury P. Djordjevic S. P. (2018). Genomic analysis of multidrug-resistant *Escherichia coli* ST58 causing urosepsis. *Int. J. Antimicrob. Agents* 52 430–435. 10.1016/j.ijantimicag.2018.06.017 29966679

[B42] Moran R. A. Hall R. M. (2018). Evolution of regions containing antibiotic resistance genes in FII-2-FIB-1 ColV-Colla virulence plasmids. *Microb. Drug Resist.* 24 411–421. 10.1089/mdr.2017.0177 28922058

[B43] Njage P. M. K. Buys E. M. (2015). Pathogenic and commensal *Escherichia* coli from irrigation water show potential in transmission of extended spectrum and AmpC β-lactamases determinants to isolates from lettuce. *Microb. Biotechnol.* 8 462–473. 10.1111/1751-7915.12234 25488608 PMC4408178

[B44] Njage P. M. K. Buys E. M. (2017). Quantitative assessment of human exposure to extended spectrum and AmpC beta-lactamases bearing *E. coli* in lettuce attributable to irrigation water and subsequent horizontal gene transfer. *Int. J. Food Microbiol.* 240 141–151. 10.1016/j.ijfoodmicro.2016.10.011 27789039

[B45] Nüesch-Inderbinen M. Zurfluh K. Peterhans S. Hachler H. Stephan R. (2015). Assessment of the prevalence of extended-spectrum beta-lactamase-producing *Enterobacteriaceae* in ready-to-eat salads, fresh-cut fruit, and sprouts from the Swiss market. *J. Food Prot.* 78 1178–1181. 10.4315/0362-028x.jfp-15-018 26038909

[B46] Oliva M. Monno R. Addabbo P. Pesole G. Scrascia M. Calia C. (2018). IS26 mediated antimicrobial resistance gene shuffling from the chromosome to a mosaic conjugative FII plasmid. *Plasmid* 100 22–30. 10.1016/j.plasmid.2018.10.001 30336162

[B47] Pietsch M. Irrgang A. Roschanski N. Brenner Michael G. Hamprecht A. Rieber H. (2018). Whole genome analyses of CMY-2-producing *Escherichia coli* isolates from humans, animals and food in Germany. *BMC Genomics* 19:601. 10.1186/s12864-018-4976-3 30092762 PMC6085623

[B48] Plessis E. M. D. Govender S. Pillay B. Korsten L. (2017). Exploratory study into the microbiological quality of spinach and cabbage purchased from street vendors and retailers in Johannesburg, South Africa. *J. Food Prot.* 80 1726–1733. 10.4315/0362-028x.jfp-16-540 28922029

[B49] Rahube T. O. Marti R. Scott A. Tien Y. C. Murray R. Sabourin L. (2014). Impact of fertilizing with raw or anaerobically digested sewage sludge on the abundance of antibiotic-resistant coliforms, antibiotic resistance genes, and pathogenic bacteria in soil and on vegetables at harvest. *Appl. Environ. Microbiol.* 80 6898–6907. 10.1128/aem.02389-14 25172864 PMC4249014

[B50] Reid C. J. DeMaere M. Z. Djordjevic S. P. (2018). Australian porcine clonal complex 10 (CC10) *Escherichia coli* belong to multiple sublineages of a highly diverse global CC10 phylogeny. *Microb. Genom.* 5:e000225. 10.1099/mgen.0.000225 30303480 PMC6487311

[B51] Reid C. J. Wyrsch E. R. Roy Chowdhury P. Zingali T. Liu M. Darling A. E. (2017). Porcine commensal *Escherichia coli*: a reservoir for class 1 integrons associated with IS26. *Microb. Genom.* 3:e000143. 10.1099/mgen.0.000143 29306352 PMC5761274

[B52] Riley L. W. (2014). Pandemic lineages of extraintestinal pathogenic *Escherichia coli*. *Clin. Microbiol. Infect.* 20 380–390. 10.1111/1469-0691.12646 24766445

[B53] Roy Chowdhury P. Charles I. G. Djordjevic S. P. (2015). A role for Tn6029 in the evolution of the complex antibiotic resistance gene loci in genomic island 3 in enteroaggregative hemorrhagic *Escherichia coli* O104:H4. *PLoS One* 10:e0115781. 10.1371/journal.pone.0115781 25675217 PMC4326458

[B54] Roy Chowdhury P. McKinnon J. Liu M. Djordjevic S. P. (2018). Multidrug resistant uropathogenic *Escherichia coli* ST405 with a novel, composite IS26 transposon in a unique chromosomal location. *Front. Microbiol.* 9:3212. 10.3389/fmicb.2018.03212 30671039 PMC6331395

[B55] Sacramento A. G. Fernandes M. R. Sellera F. P. Munoz M. E. Vivas R. Dolabella S. S. (2018). Genomic analysis of MCR-1 and CTX-M-8 co-producing *Escherichia coli* ST58 isolated from a polluted mangrove ecosystem in Brazil. *J. Glob. Antimicrob. Resist.* 15 288–289. 10.1016/j.jgar.2018.10.024 30404044

[B56] Schauss T. Glaeser S. P. Gutschow A. Dott W. Kampfer P. (2015). Improved detection of extended spectrum beta-lactamase (ESBL)-producing *Escherichia coli* in input and output samples of German biogas plants by a selective pre-enrichment procedure. *PLoS One* 10:e0119791. 10.1371/journal.pone.0119791 25799434 PMC4370489

[B57] Van Elsas J. D. Turner S. Bailey M. J. (2003). Horizontal gene transfer in the phytosphere. *New Phytol.* 157 525–537. 10.1046/j.1469-8137.2003.00697.x 33873398

[B58] Wolters B. Widyasari-Mehta A. Kreuzig R. Smalla K. (2016). Contaminations of organic fertilizers with antibiotic residues, resistance genes, and mobile genetic elements mirroring antibiotic use in livestock? *Appl. Microbiol. Biotechnol.* 100 9343–9353. 10.1007/s00253-016-7742-y 27522197

[B59] Wood J. L. Chen J. C. Friesen E. Delaquis P. Allen K. J. (2015). Microbiological survey of locally grown lettuce sold at farmers’ markets in Vancouver, British Columbia. *J. Food Prot.* 78 203–208. 10.4315/0362-028x.jfp-14-199 25581197

[B60] Wyrsch E. R. Reid C. J. DeMaere M. Z. Liu M. Y. Chapman T. A. Roy Chowdhury P. (2019). Complete sequences of multiple-drug resistant IncHI2 ST3 plasmids in *Escherichia coli* of porcine origin in Australia. *Front. Sustain. Food Syst.* 3:18 10.3389/fsufs.2019.00018

[B61] Yamaji R. Friedman C. R. Rubin J. Suh J. Thys E. McDermott P. (2018). A population-based surveillance study of shared genotypes of *Escherichia coli* isolates from retail meat and suspected cases of urinary tract infections. *mSphere* 3:e00179-18. 10.1128/mSphere.00179-18 30111626 PMC6094058

[B62] Zurfluh K. Albini S. Mattmann P. Kindle P. Nüesch-Inderbinen M. Stephan R. (2019). Antimicrobial resistant and extended-spectrum beta-lactamase producing *Escherichia coli* in common wild bird species in Switzerland. *MicrobiologyOpen* 8:e845. 10.1002/mbo3.845 31006991 PMC6855137

